# Bat lung epithelial cells show greater host species-specific innate resistance than MDCK cells to human and avian influenza viruses

**DOI:** 10.1186/s12985-018-0979-6

**Published:** 2018-04-10

**Authors:** Tessa Slater, Isabella Eckerle, Kin-Chow Chang

**Affiliations:** 1 School of Veterinary Medicine and Science, University of Nottingham, Sutton Bonington, LE12 5RD UK; 2 Geneva Center for Emerging Viral Diseases, University Hospital of Geneva Rue Gabrielle-Perret-Gentil 4, CH-1205 Geneva, Switzerland

**Keywords:** Influenza, Bats, Epithelial cells, Host resistance, Innate immunity, Inflammation

## Abstract

**Background:**

With the recent discovery of novel H17N10 and H18N11 influenza viral RNA in bats and report on high frequency of avian H9 seroconversion in a species of free ranging bats, an important issue to address is the extent bats are susceptible to conventional avian and human influenza A viruses.

**Method:**

To this end, three bat species (*Eidolon helvum*, *Carollia perspicillata* and *Tadarida brasiliensis*) of lung epithelial cells were separately infected with two avian and two human influenza viruses to determine their relative host innate immune resistance to infection.

**Results:**

All three species of bat cells were more resistant than positive control Madin-Darby canine kidney (MDCK) cells to all four influenza viruses. TB1-Lu cells lacked sialic acid α2,6-Gal receptors and were most resistant among the three bat species. Interestingly, avian viruses were relatively more replication permissive in all three bat species of cells than with the use of human viruses which suggest that bats could potentially play a role in the ecology of avian influenza viruses. Chemical inhibition of the JAK-STAT pathway in bat cells had no effect on virus production suggesting that type I interferon signalling is not a major factor in resisting influenza virus infection.

**Conclusion:**

Although all three species of bat cells are relatively more resistant to influenza virus infection than control MDCK cells, they are more permissive to avian than human viruses which suggest that bats could have a contributory role in the ecology of avian influenza viruses.

## Background

Bats (order Chiroptera) are natural reservoirs for zoonotic viruses that cause some of the deadliest diseases in humans, including filoviruses (such as Ebola and Marburg viruses), lyssaviruses, severe acute respiratory syndrome (SARS)-related coronaviruses and henipaviruses (e.g. Hendra and Nipah viruses) [1–3]. Despite being hosts to such an array of pathogens, bats generally show mild or no clinical symptoms to their presence, a phenomenon that is largely a mystery and a potential biomedical treasure trove that could offer new insights into the treatment and control of such pathogens in humans and affected animals. The lack of illness does not mean that bat cells are not infected by such viruses. Bat cells are susceptible to infections with paramyxoviruses and filoviruses [4], and show varying degree of permissiveness to virus replication, which is a pre-requisite for the hosts to acquire carrier status. Bat lung epithelial cells (TB1-Lu) of *Tadarida brasiliensis* display resistance to reovirus infection; infected cells show no cytopathic effects and rapid decline in virus production; however, low virus release is maintained for at least 2 months [5]. Murine encephalomyocarditis virus, in contrast, causes severe cytopathic damage in TB1 Lu cells, and Ebola virus shows persistent infection in such cells [5].

Recently, two novel influenza viruses, H17N10 and H18N11, were identified in bats by deep sequencing analyses (although live viruses have not been directly isolated) which have understandably caused much speculation about their zoonotic potential [6]. These viruses are, however, highly divergent from conventional mammalian and avian influenza A viruses. Chimeric virus housing the six core genes from bat H17N10 virus replicated well in human primary airway epithelial cells and mice, but poorly in avian cells and chicken embryos without further adaptation [7]. Furthermore, the chimeric bat virus failed to reassort with conventional influenza viruses in MDCK cells [7]. Bat viral ribonucleopolymerase (vRNP) complex subunits (PB1, PB1 and PA) were not functionally interchangeable with corresponding human virus-derived vRNP subunits suggesting there is limited reassortment potential between bat and human influenza viruses [8]. However, vRNP from bat H17N10 virus is able to drive with high efficiency the non-coding region of human H1N1 virus (A/WSN/1933) in vRNP minigenome reporter assays, highlighting the possibility of viable reassortment between bat and human influenza viruses [9]. Although the issue of functional reassortment between native bat and conventional influenza A viruses has not been fully resolved, its likelihood is presently considered low.

Single-cycle green fluorescent protein (GFP) reporter virus (human A/WSN/33) was variably able to infect all eleven bat cell lines, derived from seven bat species [8]. Similar number of infected cells were found among all seven bat cell lines by immunocytochemical detection of viral nucleoprotein (NP) [4]. Human virus-derived vRNP complex was shown to perform better than avian virus-derived vRNP complex in the same A/WSN/33 viral backbone at progeny virus release, based mostly on the use of TB1-Lu bat cells, which appear inherently resistant to influenza virus infection [8]. Although there is limited potential for reassortment between human and bat influenza viruses [8], *Pteropus alecto* kidney cells were able to produce reassorted progeny from human H1N1 (A/WSN/1933) and highly pathogenic avian influenza (HPAI) H5N1 (A/Vietnam/1203/04) viruses [10]. Collectively, these findings appear to indicate that bat cells are susceptible to infection with conventional mammalian and avian influenza viruses. However, we are unclear about the relative permissiveness of bat respiratory epithelial cells to conventional influenza viruses in the production of viable progeny. Although bats are not known to act as hosts for human and avian influenza viruses, the potential epidemiological significance of avian influenza virus infection in bats was highlighted by the recent discovery that around 30 out of 100 free ranging *Eidolon helvum* (fruit bats) in Ghana were serologically positive for avian H9 virus [11].

We report here on the relative susceptibility of lung epithelial cells from three diverse bat species, *T. brasiliensis* (a medium insectivorous bat)*, E. helvum,* (a large fruit bat) and *C. perspicillata* (a small mainly fruit, and insect eating bat), to avian and human influenza A viruses. We found that all three species of bat cells were more resistant than control Mardin-Darby canine kidney (MDCK) cells, in terms of reduced progeny virus production and higher cell viability, which appeared not to depend on JAK/STAT signalling. Although the three species of bat cells showed variation in resistance to infection, they were relatively more permissive to avian than human influenza viruses which could be important in the ecology of avian influenza viruses.

## Methods

### Bat and MDCK cells

*Eidolon helvum* (*E. helvum*) and *Carollia perspicillata* (C. perspic) cells were generated as described previously [12]. MDCK (ATCC CCL-34), TB1-Lu (ATCC CCL-88), *E. helvum* and C. perspic cells were cultured in DMEM-Glutamax I (high glucose) (Life Technologies) supplemented with 10% foetal calf serum and 1% penicillin streptomycin (P/S).

### Virus infection and detection

Human USSR H1N1 virus (A/USSR/77) (USSR H1N1), pandemic H1N1 2009 virus (A/California/07/2009) (pdm H1N1), low pathogenicity avian influenza (LPAI) H2N3 virus (A/mallard duck/England/7277/06), and LPAI H6N1 virus (A/turkey/England/198/09) were used. Viruses were propagated in 10-day old embryonated chicken eggs in accordance to Operation of the Animals (Scientific Procedures) Act 1986 (UK). Forty-eight hours post-infection (hpi), allantoic fluid was harvested and virus was titrated and stored at − 80 °C. Cells were washed once with phosphate-buffered saline (PBS) and infected with specified dose of virus in serum-free infection medium (Ultraculture, Lonza) supplemented with 1% P/S, 1% glutamine and 500 ng/ml tosyl phenylalanyl chloromethyl ketone (TPCK) trypsin. After 2 h of virus incubation, cells were washed three times with PBS, and incubated in fresh infection medium for a further specified period. For virus quantification, focus forming assay was performed on MDCK cells that were infected for 6 h. Cells were immunolabelled using an EnVision+ system-HRP (DAB) kit (Dako) according to the manufacturer’s instructions. Mouse monoclonal antibody (AA5H; Abcam) was used at 1 μg/ml for 40 min for viral nucleoprotein (NP) detection. Positively stained cells were visualised and counted using an inverted microscope. The average number of cells positive for NP in six wells of a 96-well plate was used to calculate infectious focus-forming units (ffu) of virus per microlitre of infection volume from which MOI dosage was derived. A virus dose of 1.0 MOI is regarded as the minimum volume of virus needed to infect each MDCK cell in a culture well as determined by NP detection at 6 hpi. All virus work was conducted in BSL-2 containment.

### Quantification of viral M-gene RNA expression

Total RNA was isolated using an RNeasy Plus minikit (Qiagen) and cDNA synthesis reaction was performed with 1 μg of total RNA using a SuperScript III first-strand synthesis kit (Invitrogen). Viral M-gene RNA expression was quantified by TaqMan real-time PCR as previously described [13, 14]. Amplification was carried out in triplicates from three biological replicates. The conditions for PCR were initial denaturation at 95 °C for 10 min followed by 45 cycles of 95 °C for 15 s, 55 °C for 30 s and 72 °C for 1 s and final cooling to 4 °C. RNA expression levels were normalised to the 18S rRNA gene.

### Host influenza virus receptors

Cells were grown on 8-well Lab-Tek II chamber slides (Nunc). Lectin labelling was performed as previously described [15]. Cells were fixed with 4% paraformaldehyde for 10 min at room temperature, after which endogenous biotin activity was blocked using a streptavidin/biotin blocking kit (Vector Laboratories). Cells were incubated with fluorescein isothiocyanate (FITC)-labelled *Sambucus nigra agglutinin* (SNA), specific for human influenza receptor type SAα2,6-Gal, and biotinylated *Maackia amurensis* agglutinin II (MAA II) (Vector Laboratories) specific for avian influenza receptor type SAα2,3-Gal overnight at 4 °C, at 10 μg/ml each. After overnight incubation, cells were washed twice with Tris-buffered saline (TBS) and subsequently incubated with streptavidin-Alexa Fluor 594 conjugate (Invitrogen) at room temperature for 2 h. Finally, cells were washed three times with TBS and mounted with ProLong Gold antifade reagent with 4′,6′ diamidone-2-phenylindole (DAPI) (Invitrogen). Neuraminidase derived from *Clostridum perfringens* (11,585,886,001; Roche) was used at 0.05 U/ml in culture medium for 4 h at 37 °C for the collective removal of SA receptors [16].

### Endosomal uptake of siRNA

Cells were transfected using the Viromer Blue system (Lipocalyx) with a SignalSilence Control siRNA (Fluorescein Conjugate) (#6201, Cell Signaling Technology) according to manufacturer’s instructions.

### Flow cytometry for quantification of cell viability

Cell viability, based on entry of fluorescent dye into cells with compromised cell membranes, was quantified using a LIVE/DEAD Fixable far red fluorescent kit (L10120; Life Technologies) in a BD FACS CANTO II flow cytometer (BD Biosciences). Data analysis was carried out using the Kaluza Analysis software (Beckman Coulter). A heat-killed control, subjected to 60 °C for 20 min, and uninfected control were used to determine the fluorescence threshold between viable and dead cells.

### Western blotting

Radioimmunoprecipitation assay (RIPA) buffer (Santa Cruz) supplemented with 1% phenylmethylsulfonyl fluoride (PMSF) (Santa Cruz), 1% cocktail inhibitor and 1% sodium orthovanadate (Santa Cruz) was used to lyse cells. Bio-RAD protein assay was used to determine protein concentration (Bio-Rad). Primary antibodies used were mouse anti-viral NP (at 1:3000 dilution; PA5–32242, Pierce), goat anti-viral PB1 (at 1:10000 dilution; 17,601, Santa Cruz), goat anti-viral M1 (at 1:2000 dilution; ab20910, Abcam), mouse anti-β-actin (at 1:10000 dilution; A5316, Sigma) and secondary antibodies used were donkey anti-goat IgG (at 1:10000 dilution; sc-2020, Santa Cruz) and goat anti-mouse IgG (at 1:1000 dilution; HAF007, R&D Systems).

### Inhibitors of JAK-STATsignalling

Pyridone 6 (Merck), a JAK inhibitor, was applied at 5 μM [17] to cells for 20 h prior to infection at 37 °C. Cells were rinsed twice with PBS and then infected with USSR H1N1 virus at 1.0 MOI. DMSO treated cells were infected as controls. After 2 h infection, cells were rinsed three times with PBS and fresh infection medium was added with corresponding inhibitor and incubated for a further 22 h before virus titration on MDCK cells using spun supernatants from infected cells.

### Statistical analysis

Statistical analysis was performed using GraphPad Prism 6 (GraphPad Software). Student’s t-test, one-way ANOVA and two-way ANOVA were used as appropriate. *P* values < 0.05 were considered to be significant.

## Results

### Bat respiratory epithelial cells were more resistant to influenza virus infection than MDCK cells but were relatively more permissive to avian than human virus strains

**Fig. 1:**
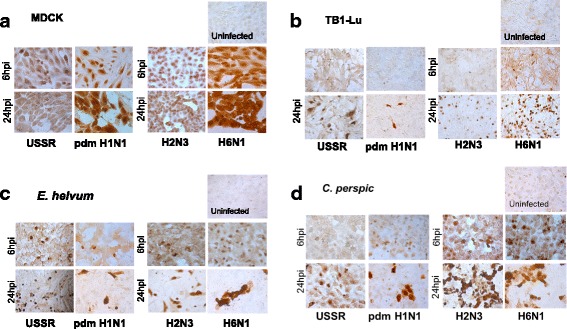
Bat respiratory epithelial cells were less susceptible than MDCK cells to influenza A virus infections but showed differential susceptibility between human and avian influenza viruses. Epithelial cells of *T. brasiliensis* (TB1-Lu), *E. helvum*, C. perspic and control MDCK cells were separately infected with human (USSR H1N1 and pdm H1N1) and avian (H2N3 and H6N1) viruses at 1.0 MOI for 6 h and 24 h, and immunostained (brown) for viral NP. Expectedly, MDCK cells were extensively infected showing nuclear localisation of NP at 6 hpi, and widespread cytoplasmic spread of NP at 24 hpi with each virus type (**a**). TB1-Lu cells, by contrast, were not readily infected by human or avian viruses; even at 24 hpi only a limited number of cells showed intranuclear NP localisation (**b**). *E. helvum* and C. perspic cells showed differential infection susceptibility between human and avian viruses. Cells from the two bat species, like TB1-Lu cells, were not readily infected with human viruses (USSR H1N1 or pdm H1N1 virus) such that relatively few cells showed intranuclear NP localisation at 24 hpi (**c** and **d**). However, *E. helvum* and C. perspic cells were extensively infected by 6 hpi with avian viruses (H2N3 or H6N1 virus), and by 24 hpi exhibited extensive cell loss and cytoplasmic spread of NP (**c** and **d**)

Despite speculation that bats may be potential hosts for conventional influenza viruses, few studies have examined the infectious relationship between influenza viruses and bats. Lung epithelial cells derived from three bat species, *T. brasiliensis* (TB1-Lu) *E. helvum* and *C. perspicillata* (C. perspic), were subjected to virus infection using two LPAI viruses, H2N3 and H6N1 virus, and two human influenza viruses, USSR H1N1 and pdm H1N1 virus, at a multiplicity of infection (MOI) of 1.0, based on focus forming assays. MDCK cells were used as a comparative control cell line due to their use as a reference cell line for influenza A virus titration [18, 19]. MDCK and bat cells were immunostained for viral NP at 6 and 24 hpi (Fig. 1).

At 6 hpi, intranuclear viral NP was extensively detected in MDCK cells infected with each of the four viruses and at 24 hpi NP presence had extended into the cytoplasm (Fig. 1a). Similar extensive pattern of infection was not observed with bat cells. TB1-Lu cells were not readily infected by either human or avian viruses. At 6 hpi, there was little or no detection of NP; at 24 hpi, NP was found only in a limited proportion of nuclei (Fig. 1b). In *E. helvum* cells infected with LPAI viruses (H2N3 and H6N1), NP was extensively detected by 6 hpi (Fig. 1c). By 24 hpi, NP was detected in the cytoplasm accompanied by widespread detachment of cells. In contrast, in *E. helvum* cells infected with human viruses (USSR H1N1 and pdm H1N1), NP was only detected in a small proportion of cells even at 24 hpi suggesting that they were less infected with human than avian influenza viruses. A similar infection pattern was observed in C. perspic cells. At 6 hpi with avian viruses, most nuclei of C. perspic cells were strongly labelled for NP and by 24 hpi, cytoplasmic NP was evident with extensive cell loss (Fig. 1d). With human USSR or pdm H1N1 virus, limited number of C. perspic cells were infected even at 24 hpi with NP largely localised to the nuclei. In summary, all three species of bat cells infected with human and avian influenza viruses showed much less viral NP production than correspondingly infected MDCK cells, with TB1-Lu cells producing the least viral NP. Relatively, *E. helvum* and C. perspic cells showed greater NP production and cell loss at 24 hpi when infected with avian than with human viruses.

**Fig. 2:**
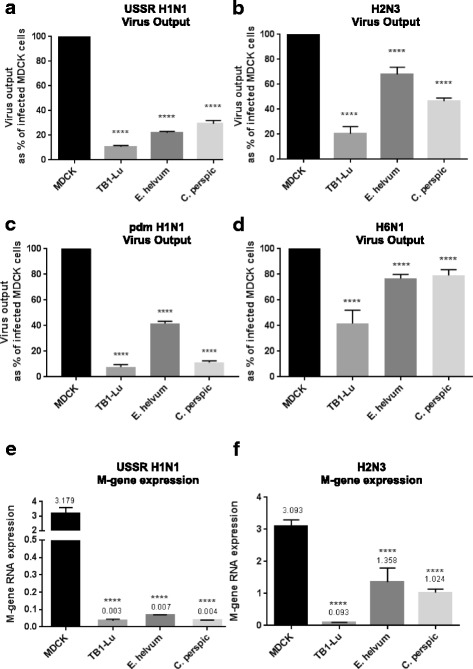
Infected bat cells produced significantly less progeny influenza viruses and viral M-gene RNA than correspondingly infected MDCK cells. Cells of all three bat species (TB1-Lu, *E. helvum* and C. perspic) and MDCK cells were infected with human or avian viruses at 0.5 MOI (based on focus forming assays) for 24 h. Supernatants were titrated on MDCK cells in 6 h focus forming assays to quantify progeny virus release. Infected bat cells of all three species produced significantly less viable virus than correspondingly infected MDCK cells (**a**-**d**). TB1-Lu cells released the least progeny virus among the three bat species. Furthermore, proportionally more progeny avian (H2N3 and H6N1) than human (USSR and pdm H1N1) viruses were produced from each species of bat cells. Results shown are the combined results of three independent experiments. Typically virus output from infected MDCK cells is in the region of 200 ffu/μl (**a**-**d**). Extracted total RNAs were quantified for viral M-gene expression normalised to 18 s rRNA (**e** and **f**). Cells of all three bat species produced significantly less viral M-gene RNA than MDCK cells with each virus. Each species of bat cells also generated more M-gene RNA from avian H2N3 virus than from USSR H1N1 virus infection. Results are representative of three experimental repeats

Viable progeny virus output from all three species of bat cells was significantly less (*P* ≤ 0.0001) than from correspondingly infected (at 0.5 MOI for 24 h) MDCK cells (Fig. 2a-d). Notably, avian H2N3 and H6N1 influenza viruses (Fig. 2b and d) generated proportionally more progeny virus than human USSR H1N1 and pdm H1N1 viruses (Fig. 2a and c) for each type of bat cells. Among the three species, TB1-Lu cells clearly produced the least progeny viruses (Fig. 2a-d). Predictably, the pattern of viral M-gene RNA expression (normalised to 18 s rRNA) from infected MDCK and bat cells closely matched the pattern of virus output (Fig. 2e and f). Bat cells expressed less M-gene RNA (*P* ≤ 0.0001) than correspondingly infected MDCK cells. Furthermore, avian H2N3 virus conferred higher M-gene expression than corresponding human USSR H1N1 virus infection in each bat species. Collectively, these results indicate that all three species of bat cells are more resistant than MDCK cells to influenza virus infection of which TB1-Lu cells are most resistant, and that *E. helvum* and C. perspic cells appear more replication permissive and exhibit greater cytopathogenicity to avian than to human influenza viruses.

### Lack of human sialic acid α2,6 linkage receptor contributed to host resistance in TB1-Lu cells

**Fig. 3:**
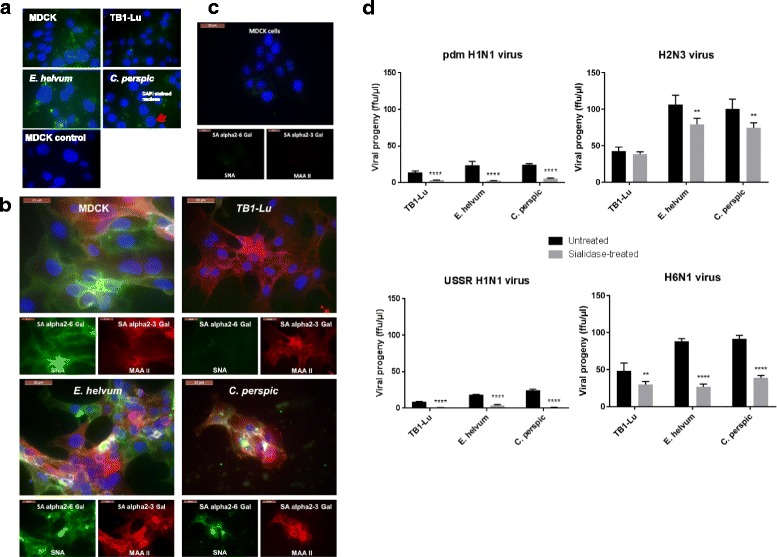
Host SA receptors contribute to influenza virus entry into bat cells. **a** TB1-Lu cells exhibited the least uptake of FITC-labelled siRNA in comparison with MDCK, *E. helvum* and C perspic cells. Respiratory epithelial cells from the three bat species and MDCK cells were transfected with FITC-labelled siRNA with the use of Viromer Blue reagent which mimics natural influenza viral entry and membrane fusion. Fluorescence imaging at 4 h showed detectable uptake of siRNA (green spots) in MDCK, *E. helvum* and C. perspic cells. However, TB1-Lu cells barely showed any fluorescence. Mock transfected MDCK cells showed complete absence of fluorescence. **b** *E. helvum* and C. persic but not TB1-Lu cells expressed human and avian SA receptors. Bat and MDCK cells were subjected to binding by plant lectins SNA and MAA II for the detection of human SAα2,6 Gal (green) and avian SAα2,3 Gal (red) receptors respectively. Avian SAα2,3 Gal and human SAα2,6 Gal receptors were readily found in all cell types except for TB1-Lu cells where SAα2,6 Gal receptor was barely detected. Nuclei were counterstained with DAPI (blue). **c** SA receptors were effectively removed from MDCK cells treated with a broad spectrum neuraminidase derived from *Clostridium perfringens*. Post-treatment, there was hardly any detection of SA receptors using MAA II and SNA lectins. **d** Collective removal of SA receptors with neuraminidase resulted in a marked reduction in progeny virus production from all three bat species. All results shown are representative of three experimental repeats

The transfection of bat cells with FITC-labelled siRNA using Viromer blue reagent (Lipocalyx), designed to mimic natural influenza viral entry and membrane fusion [20], showed reduction in endosomal uptake of siRNA by TB1-Lu relative to *E. helvum*, C. perspic and MDCK cells (Fig. 3a). This reduced uptake by TB1-Lu cells prompted us to examine the distribution of host sialic acid (SA) receptors on bat cells. Entry of conventional influenza virus into a host cell requires viral hemagglutinin binding to SA receptors on the cell surface. Human influenza A viruses are adapted to SA receptors with α-2,6 linkage whereas avian virus strains are more adapted to SA receptors with α-2,3 linkage [21–23]. We examined the expression and spatial distribution of SAα2,3-GalG(1–3)GalNAc and SAα2,6-Gal receptors in the three species of bat and MDCK cells by binding with plant lectins MAA II and SNA, respectively, to determine if they could potentially account for host species differences in influenza virus susceptibility. Both SAα2,3-Gal and SAα2,6-Gal receptors were extensively detected in MDCK, *E. helvum* and C. perspic cells (Fig. 3b). However, in TB1-Lu cells the avian SAα2,3-Gal receptor was clearly present; human SAα2,6-Gal receptor was barely detected. The lack of a major receptor type in TB1-Lu cells could contribute to host resistance (Figs. 1 and 2) by reducing cell entry of influenza viruses.

To further explore the functional impact of SA receptors on influenza virus infection, SA receptors were collectively removed from the cell surface using a generic neuraminidase derived from *Clostridium perfringens*, as previously described [16] (Fig. 3c)*.* In all cell types, the removal of SA receptors resulted in sharp decrease in virus output (Fig. 3d) indicating their importance in virus infection. However, despite extensive removal of SA receptors, there remained some virus production suggesting the presence of an alternative SA-independent pathway for virus entry. In summary, SAα2,3-Gal and SAα2,6-Gal receptors appear important in bat cells for entry of conventional influenza A viruses, and the deficiency of SAα2,6-Gal receptor in TB1-Lu cells could be an added factor of host resistance to entry of human influenza viruses in this cell type.

### Bat cells appeared more viable than MDCK cells infected with influenza viruses

**Fig. 4:**
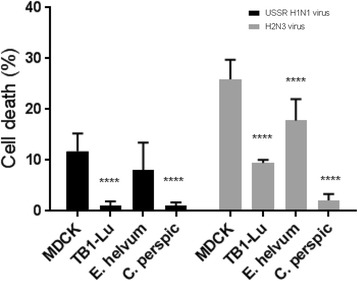
Influenza virus infected bat cells appeared more viable than correspondingly infected MDCK cells. Cells were infected at 0.5 MOI with human USSR virus or avian H2N3 virus for 24 h and subsequently analysed by flow cytometry for cell membrane integrity. Avian H2N3 virus elicited higher level of cell death than corresponding USSR virus for each cell type. In addition, with the exception of USSR virus infected *E. helvum* cells, each virus caused significantly higher cell death in MDCK than bat cells. Inset shows the same results presented as comparisons of cell death between virus types. Results shown are representative of three experimental repeats

Flow cytometry was used to determine cell viability through the detection of incorporated fluorescent dye in cells with damaged cell membranes. All three species of bat cells infected with avian H2N3 virus were more viable than correspondingly infected MDCK cells (*P* ≤ 0.0001) (Fig. 4). Similar results were obtained with the use of human USSR virus but less cell damage to each cell type was noted (Fig. 4). With the exception of C. perspic cells, avian H2N3 virus caused greater increase in cell death than human USSR virus in all other cell types which was consistent with the earlier findings in virus output and viral M-gene RNA expression that bat cells were more permissive to avian than human influenza viruses. Among the three bat cell lines, however, *E. helvum* cells showed the highest percentage of cell death whereas C. perspic cells were most viable post-infection with 1% and 2.1% cell death from infection with USSR H1N1 virus and H2N3 virus respectively.

**Fig. 5:**
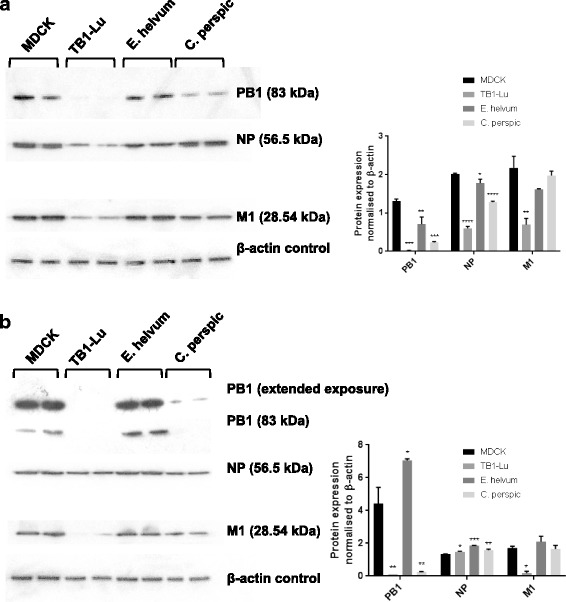
Variation in viral protein production between infected bat cells. All three species of bat cells and MDCK cells were infected with human USSR H1N1 (**a**) or avian H2N3 (**b**) virus at 0.5 MOI for 24 h for the detection of viral PB1, NP and M1 along with detection of β-actin as loading control. Differential viral protein expression of PB1, NP and M1 was evident between the three bat cell types for each virus (**a** and **b**). All three viral proteins were strongly expressed in MDCK and *E. helvum* cells with each virus. USSR H1N1 virus infected TB1-lu cells showed weak expression of each viral protein; similarly avian H2N3 virus infected TB1-Lu cells showed faint presence of PB1 and M1 proteins. Infected C. perspic cells displayed an intermediate pattern of viral protein expression that was between that of correspondingly infected *E. helvum* and TB1-Lu cells. Insets are corresponding densitometric quantification of Western blotting results. Significance indicated is in relation to corresponding MDCK cells

Western blotting was conducted to characterise viral protein expression in the three species of bat cells infected with avian H2N3 or human USSR H1N1 virus (Fig. 5). There was wide variation in viral protein production between infected cells (Fig. 5a and b). All three viral proteins (PB1, NP and M1) were clearly expressed in MDCK and *E. helvum* cells with each virus infection. USSR H1N1 virus-infected TB1-Lu cells showed weak expression of each viral protein; similarly, avian H2N3 virus-infected TB1-Lu cells showed faint production of PB1 and M1 protein. Infected C. perspic cells displayed an intermediate pattern of viral protein expression that was between that of correspondingly infected *E. helvum* and TB1-Lu cells. In summary, the three species of infected bat cells were more viable than correspondingly infected MDCK cells, with relatively higher cell death from avian H2N3 virus than human USSR virus infection. With the possible exception of TB1-Lu cells, variation in expression of viral protein levels alone could not fully account for the differences in virus output or cell viability observed between the species of bat cells which suggest the possible presence of translational/post-translational inhibition of virus in bat cells as another level of host resistance.

### The JAK-STAT pathway did not appear to play a key anti-viral role in bat cells

**Fig. 6:**
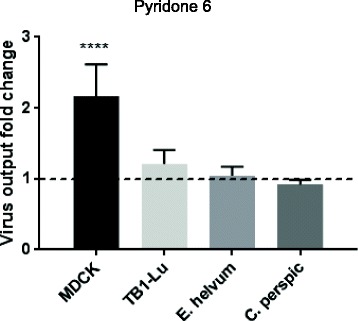
Inhibition of the JAK/STAT pathway did not appear to affect virus output from infected bat cells. Cells were treated with 5 μM pyridone 6 (JAK inhibitor), for 20 h prior to infection with human USSR virus at 0.5 MOI. There was no notable difference in virus output between treated TB1-Lu, *E. helvum* and C. perspic cells, and their untreated counterparts. Pyridone 6 treated MDCK cells, however, showed a 2-fold increase in virus output. Results shown are representative of three experimental repeats

The JAK-STAT pathway is a key signalling pathway of type I interferons, cytokines and growth factors in the regulation of immune functions, in particular in anti-viral responses [24]. To assess the role of JAK-STAT signalling during influenza virus infection, the three species of bat cells were treated with 5 μM pyridone 6 (JAK inhibitor) [17] for 20 h prior to infection overnight with USSR H1N1 virus at 0.5 MOI. Despite the relatively substantial dose of JAK inhibitor used (e.g. IC_50_ for murine JAK1 is only 15 nM), treated bat cells showed no significant difference in virus output compared with their untreated counterparts (Fig. 6). By contrast, virus output of control MDCK cells increased by more than 2-fold when JAK was inhibited (Fig. 6). The available protein sequences of JAK1 to 3 from several other bat species have on average about 90% homology with their canine and porcine counterparts (Table 1) suggesting that pyridone 6 at sufficiently high dose is likely to target bat JAKs. Thus, it appears that the JAK-STAT pathway in the three species of bat cells is not critical in the control of influenza virus production.

**Table 1:** Protein sequence similarities of JAK1 to 3 between different bat species and other mammalian species (canine and porcine)

	Percentage identity to *Canis lupus familiaris* (%)			Percentage identity to *Sus scrofa* (%)		
	JAK1	JAK2	JAK3	JAK1	JAK2	JAK3
*Myotis brandtii*	91.65	91.23	85.48	91.16	91.62	86.01
*Myotis lucifugus*	89.38	92.41	84.86	89.29	92.95	85.28
*Rhinolopus sinicus*	88.47	90.89	85.94	89.54	91.99	85.70
*Eptesicus fucus*	92.39	91.00	88.63	92.30	92.83	88.43
*Hipposideros armiger*	88.63	91.62	86.28	89.22	92.35	86.62
Average similarity	90.10	91.43	86.24	90.30	92.35	86.41

## Discussion

In this study, we attempted to address the issue of relative susceptibility of bats to conventional human and avian influenza A viruses which could have fundamental and epidemiological importance given their widespread global distribution. The recent finding of 30% of free ranging *E. helvum* (large fruit bats) in Ghana to be serologically positive for avian H9 virus highlights the possibility of transmission of avian influenza viruses into bats [11]. We found that the lung epithelial cells of three diverse bat species, *T. brasiliensis* (a medium insectivorous bat), *E. helvum* and *C. perspicillata* (a small mainly fruit bat) were consistently more resistant to avian and human influenza A viruses than correspondingly infected control MDCK cells in terms of reduced progeny virus production, less infected cells and greater cell viability. Interestingly, between avian (H2N3 and H6N1) and human (USSR H1N1 and pdm H1N1) viruses, bat cells, in particular *E. helvum* and C. perspic, infected with avian viruses were more permissive to virus production and showed greater cytopathogenicity than those infected with human viruses.

There was variation in resistance to influenza virus infection between the three species of bat cells. TB1-Lu cells were most resistant among the three species, producing the least number of infected cells and progeny viruses. Both host SAα2,3-Gal and SAα2,6-Gal receptors appear important in all bat cells for entry of conventional influenza A viruses in that their removal by sialidase treatment led to significant reduction in virus output. The weak presence of SAα2,6-Gal receptor in TB1-Lu cells could account in part for host resistance to the entry of human influenza viruses. There were differences in the expression of viral proteins (PB1, M1 and NP) between the three bat cell types separately infected with avian H2N3 and human USSR H1N1 virus. Such differences in viral protein expression could not readily account for the differences in virus output or cell viability observed between the species of bat cells but hint at the possible presence of translational/post-translational mechanisms of virus inhibition in bat cells as added layers of host innate resistance.

Given that bats play hosts to a diverse range of deadly zoonotic viruses often without serious clinical consequences to themselves, it is reasonable to assume that there are generic innate immune responses in bats that are effective across different viral pathogens. Insights into such processes could provide invaluable basic knowledge for the control and treatment of lethal human infections transmitted by bats. The JAK-STAT pathway is a major signalling pathway of type I interferons and cytokines in the transcriptional activation of anti-viral responses. Based on the use of pyridone 6 (JAK inhibitor), we found that the JAK-STAT pathway in the three species of bat cells appeared not to be critical in the control of influenza virus production. This observation is consistent with the recent finding that certain bat cells have a dampened interferon response due to the replacement of the highly conserved serine residue (S358) in STING, an essential adaptor protein in multiple DNA sensing pathways [25]. Additional work is needed to further assess (chemically and genetically) the anti-viral role of JAK/STAT signalling in bat cells. The unavailability of bat species-specific reagents is currently a major research bottleneck hampering progress in the area. There are obvious needs for bat species-specific gene sequences and antibodies to be able to conduct quantitative PCR and Western blotting to detect members of the innate immunity such as specific interferons, cytokines and their corresponding responsive gene products. The NF-κB pathway has a complex relationship with influenza A virus. Inhibition of NF-κB signalling in murine and human respiratory epithelial cells has been shown to reduce both virus replication and production of pro-inflammatory cytokines following avian and human influenza virus infections [26, 27]. However, for the wealth of evidence that supports NF-κB as a pro-viral agent in the promotion of influenza virus propagation, there is credible evidence to show that NF-κB is also a mediator of inflammatory and anti-viral responses [28–30]. We speculate that the NF- κB pathway could be functionally more important in bat cells than the JAK/STAT pathway.

## Conclusion

In conclusion, all three species of bat cells are more resistant than control MDCK cells to conventional influenza viruses and are relatively more permissive to avian than human viruses which suggest that bats could have a contributory role in the ecology of avian influenza viruses.

## Abbreviations

C. perspic: *Carollia perspicillata*
*E. helvum*: *Eidolon helvum*
LPAI H2N3: low pathogenicity avian influenza H2N3 (A/mallard duck/England/7277/06)
LPAI H6N1: low pathogenicity avian influenza H6N1 (A/turkey/England/198/09)
MAA II: *Maackia amurensis agglutinins*
MDCK: Madin-Darby canine kidney
pdm H1N1: pandemic H1N1 2009 (A/California/07/2009)
SA: sialic acid
SNA: *Sambucus nigra agglutinin*
TB1-Lu: *Tadarida brasiliensis* lung epithelial cells
USSR H1N1: human USSR H1N1 (A/USSR/77)
